# Interactions of Meibum and Tears with Mucomimetic Polymers: A Hint towards the Interplay between the Layers of the Tear Film

**DOI:** 10.3390/ijms22052747

**Published:** 2021-03-09

**Authors:** Petar Eftimov, Norihiko Yokoi, Ana M. Melo, Philippe Daull, Georgi As. Georgiev

**Affiliations:** 1 Department of Cytology, Histology and Embryology, Faculty of Biology, St. Kliment Ohridski University of Sofia, 8 Dragan Tzankov Blvd., 1164 Sofia, Bulgaria; peftimov@uni-sofia.bg; 2 Department of Ophthalmology, Kyoto Prefectural University of Medicine, Kyoto 602-8566, Japan; nyokoi@koto.kpu-m.ac.jp; 3 Institute for Bioengineering and Biosciences, Instituto Superior Técnico, Universidade de Lisboa, 1649-004 Lisbon, Portugal; anammelo@tecnico.ulisboa.pt; 4 Novagali Innovation Center, Santen SAS, 91058 Evry, France; philippe.daull@santen.com

**Keywords:** mucomimetic polymers, cross-linked hyaluronic acid, tear film, ferning patterns, human meibum, surface properties

## Abstract

Recent clinical findings suggest that mucomimetic polymers (MMP) can alter not only the texture of the aqueous tear but also the spreading and structure of the tear film (TF) lipid layer, thereby allowing for their synchronized performance in vivo. Thus, we aimed to evaluate in vitro (i) the capability of pharmaceutically applicable MMP to ensure the formation of post-evaporative ferning patterns (a characteristic feature of the “healthy” tear colloid) and (ii) the MMP interactions with human meibum films accessed in the course of blink-like deformations via Langmuir surface balance and Brewster angle microscopy (BAM). Four MMP were used- hyaluronic acid (HA), cross-linked hyaluronic acid (CHA), carboxymethyl cellulose (CMC) and gellan gum (GG)- at the concentrations of 0.0001%, 0.001%, 0.01%, 0.05% and 0.1%. Significant differences were observed in the MMP fern formation capability: CHA (≥0.001%) > HA (≥0.01%) = CMC (≥0.01%) > GG (≥0.05%). All MMP affected the spreading of meibum, with BAM micrographs revealing thickening of the films. CHA was particularly efficient, showing concentration-dependent enhancement of tear ferning and of meibomian layer structure, surfactant properties and viscoelasticity. Thus, endogenous and exogenous MMP may play key roles for the concerted action of the TF layers at the ocular surface, revealing novel routes for TF-oriented therapeutic applications.

## 1. Introduction

Tear film (TF) represents a composite wetting film composed by tear film lipid layer (TFLL) at the air/tear surface and underlying aqueous tear (AT) situated over the glycocalyx of the corneal epithelium [1,2]. When some of the TF layers are quantitatively or qualitatively impaired, this results in premature (<10 s after eye opening) rupture of TF at the ocular surface and the onset of dry eye syndrome (DES). DES is the most prevalent public health ophthalmic disease, affecting the quality of life of 10–30% of the human population worldwide [3], and exerting major socioeconomic burden on the developed world societies.

Recently, tear film lipid layer has received a lot of attention as it was found that more than 80% of the dry eye sufferers report symptoms of meibomian gland dysfunction (MGD), a condition associated with qualitative and quantitative abnormalities in the TFLL [3]. This is because TFLL, a maze of 236 lipid species distributed among nine molecular classes [4], is actually built primarily (>95%) by the meibomian gland secretion (MGS, or simply meibum): a composite lipid-rich mixture that may also contain up to 22 wt% non-lipid components (proteins, salts, polysachharides) [5,6,7]. Meibomian lipids are composed of >90% non-polar lipids (NPL, primarily wax and sterol esters and triacylglycerols) and <10% polar amphiphilic lipids (PL, namely (*O*-acyl)-ω-hydroxy fatty acids (OAHFA) and some phospholipids) [8,9]. MGS was found to form a thick viscoelastic duplex film composed of a monomolecular layer of amphiphilic polar lipids at the aqueous interface and a NPL stratum of generally unstructured lipophilic suspension situated on top and facing the air [10,11].

If the importance of MGS for the stabilization of the air/tear surface is well recognized, the potential impact of hydrophilic polymers dissolved in AT, i.e., endogenous tear secretory mucins (MUC5AC) and exogenous mucomimetic polymers (delivered via pharmaceutical compositions), on the TF surface properties remains largely overlooked [12]. This is easy to explain as at physiological concentrations, these polymers have no surface activity and were thought to primarily contribute to the shear thinning properties and to the “tensile strength” of human tears.

However, recently, in vitro studies suggested that mucomimetic polymers (MMP) containing long polyanionic polysaccharide moieties (e.g., hyaluronic acid) emulating the sialic acid residues of secretory mucin glycoproteins, as well as MUC5AC itself [13,14], may modulate the spreading of meibomian and tear lipid films at the air/water surface. This is achieved probably by electrostatic, H-bonding and hydrophobic interactions of the MMP with the headgroups of meibomian PL [13,15]. Such findings suggest that TFLL and endogenous and exogenous MMP may interact at the physiologically relevant timescale of seconds (H-bonding is a nanoseconds event), thus allowing for synchronous concerted action between the constituent of TFLL and AT. The impact of mucin-like polymers on TFLL was clinically demonstrated by the increase of the thickness of TFLL of lipid-deficient patients, from <60 to 75 nm, 15 min post-instillation of 0.1% HA [16], and by the similar effect of 3% diquafosol ophthalmic solution (strong MUC5AC secretatgogue) on the TFLL in healthy human eyes for more than 1 h post-instillation [17].

Thus, the interactions of MMP with meibomian and tear films deserves further study as it may provide information for the fundamental mechanisms relevant to the functionality of the tear film and may result in the selection of promising polymers for further pharmaceutical implementation. Therefore, the aim of our work was to evaluate (i) the capability of pharmaceutically applicable MMP to ensure the formation of post-evaporative ferning patterns (mucin-enriched finely structured precipitates formed after evaporation of “healthy” tear microdroplets over glass substrate [1]), on their own and when supplemented into human tears, as well as (ii) their interactions with human meibum films. Four pharmaceutically applicable polymers were used—hyaluronic acid (HA), cross-linked hyaluronic acid (CHA), carboxymethyl cellulose (CMC) and gellan gum (GG)—at the concentrations of 0.0001%, 0.001%, 0.01%, 0.05% and 0.1% [12,18]. This range was chosen as typically, the concentration of MMP in eyedrops is 0.1–0.3%. Thus, it can be the highest polymer concentration attained at the ocular surface immediately at instillation, which then instantaneously gets diluted due to the rapid aqueous tear turnover [18]. Thus, the therapeutic range of mucomimetic polymer concentrations is reasonably estimated to be in the gamut of 0.0001–0.1% [1,17].

The formation of structured ferning patterns after the evaporation of tear microdroplets is a characteristic feature of the “healthy” tear colloid and a reliable indication for the mucomimeticity and for the therapeutic potential of polymer solutions [19]. The interactions between MMP and meibum at the air/water interface were examined in vitro at blink-like compression/expansion of the film area by Langmuir surface balance. Surface pressure (*π*)-area (A) isocycles and stress relaxations were used to assess the sample’s dilatational elasticity and reorganization during area cycling. Films’ morphology was monitored by Brewster angle microscopy (BAM).

## 2. Results

### 2.1. Ferning Patterns of Polymer Solutions, Pure and Included in Human Tears

**Figure 1:**
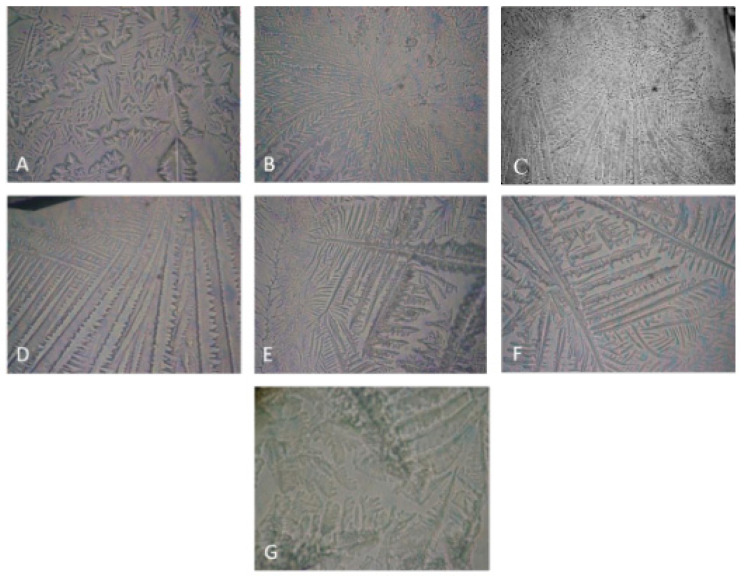
Ferning patterns observed after evaporation of microdroplets of mucomimetic polymers (MMP) solutions. (**A**) CHA 0.001%, (**B**) CHA 0.01%, (**C**): HA 0.01%, (**D**) HA 0.1%, (**E**) CMC 0.01%, (**F**) CMC 0.1%, (**G**): GG 0.05%. The field of view is 0.8 mm × 0.8 mm.

A very characteristic property rendered by the balance of MUC5AC quality and content and the ionic strength of AT is the capability of tear droplets to precipitate to a fine-structured ferning pattern after the evaporation of the tear water content [19,20]. Thus, a suitable MMP should be able to form ferning patterns on its own and to support the ones of “healthy” human tears when supplemented into them. The capability of the polymers to yield structured post-evaporative ferns is presented in Figure 1.

Significant differences were observed in the capability of MMP alone to form ferning patterns, both in terms of post-evaporative precipitates texture and minimal concentration for fern formation: CHA (≥0.001%) > HA (≥0.01%) = CMC (≥0.01%) > GG (≥0.05%). As can be seen, CHA displayed formation of structured post-evaporative crystallites at a concentration which was an order of magnitude lower compared to the rest of the MMP. At further increase of MMP concentration above the minimal one for fern formation, the density of the ferns (the number of ferns per unit area) increased, and also, their structure changed. It is interesting to note that micrograph D (HA 0.1%) in Figure 1, in addition to being distinct from all others in this figure, does not show fine patterns and has minimal branching. Also, above certain MMP concentrations, it was not possible to reliably detect further changes in the ferns’ morphology; however, due to the qualitative nature of the phenomenon, it was not further analyzed. It is assumed that the higher the ferning pattern propensity, the higher the mucomimeticity of the polymer and its capability to favorably interact with the endogenous tear MUC5AC and to enhance the functionality of the AT [19,20]. Thus, CHA shows very promising behavior by being able to form ferns at a concentration an order of magnitude lower than the rest of the polymers. It is notable that GG was not able to form fine-structured patterns even at the highest concentration used.

**Figure 2:**
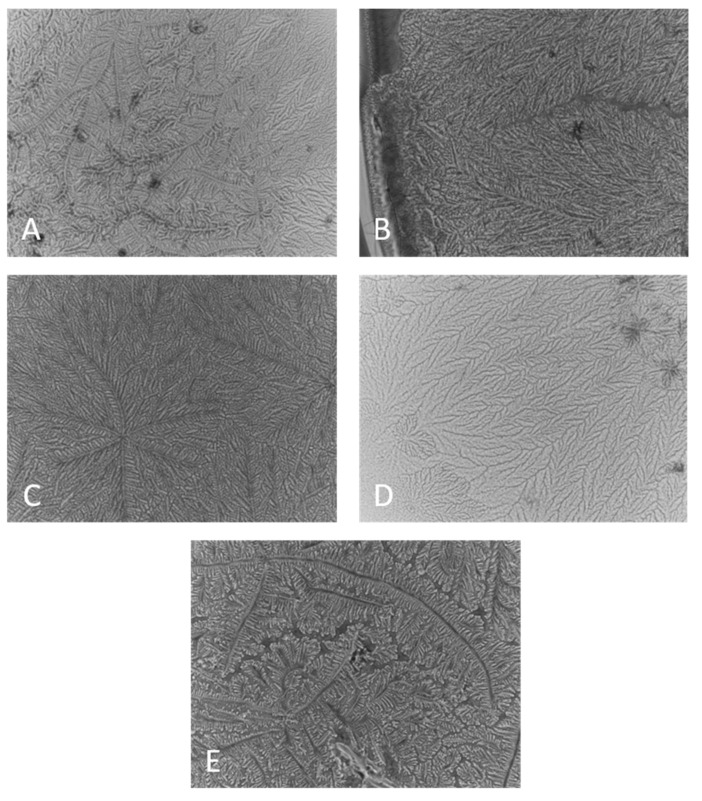
Ferning patterns observed after evaporation of microdroplets of human tears, alone and in the presence of MMP. (**A**) Pure tear, (**B**) Tears + 0.05% CHA, (**C**): Tears + 0.05% HA, (**D**) Tears + 0.05% CMC, (**E**) Tears + 0.05% GG. The field of view is 0.8 mm × 0.8 mm.

When the MMP were supplemented to real human tears (Figure 2), it can be seen that all MMP, when mixed with tears, enhanced the formation of fine-structured post-evaporative ferning pattern. The exception was GG, where cluttered-like precipitates were observed visually distinct compared to the fine-micro-structured ferning patterns observed for the rest of the tear/MMP samples.

It is well known that after evaporation, healthy tears precipitate to continuous finely structured ferns (Figure 2A). In the case of dry eye pathology, the fern formation is obstructed, and they become unstructured and patchy, with discontinuity increasing proportionally to the severity of dry eye [1]. It is interesting that recently [19], it was reported that in mild DES, the ferns become thicker and composed of bulkier aggregates, partially similar to the ones in Figure 2E. At the same time, it should be kept in mind that both clinically utilized ferning pattern grading scales (of Rolando and of Masmali) [19] rely on qualitative visual estimation of the patterns, which limits the possibility for quantitative comparison and interpretation of such results, although they remain useful for qualitative probing of tear/pharmaceutical interactions.

### 2.2. Effect of MMP on the Surface Pressure/Area Isotherms of Meibomian Films

#### 2.2.1. Effects of HA, CMC and GG

**Figure 3:**
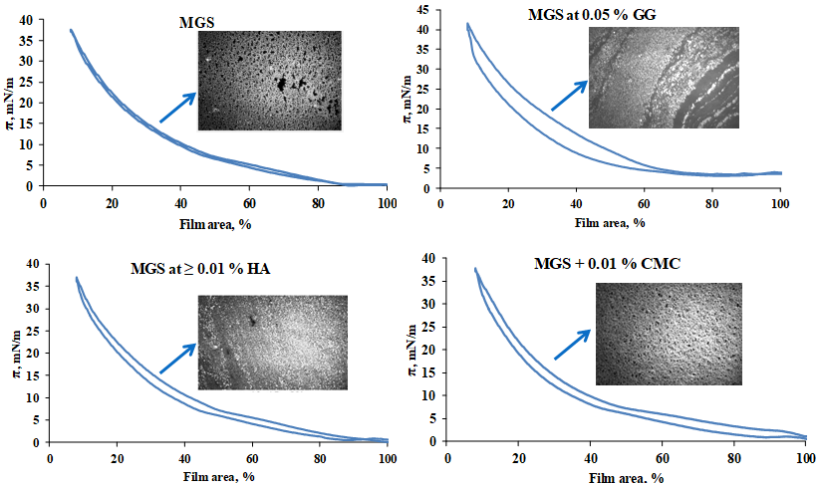
Surface pressure (mN/m)/area (%) isocycles and Brewster angle microscopy (BAM) morphologies (image width 500 µm) of meibomian films at saline solution subphase, pure and in the presence of MMP.

The compression/expansion surface pressure/area isocycles and characteristic BAM micrographs are shown (Figure 3) of MGS films over saline solution subphase, pure or in the presence of HA, CMC and GG.

HA and CMC enhance the spreading of the meibomian films, as manifested by a more uniform and thick (i.e., bright) lipid layer structure, visualized with Brewster angle microscopy, but otherwise have modest impact on MGS surface properties. It can be seen that MGS, alone and in the presence of HA and CMC, preserves high isotherm reversibility (*R_v_* = 99% and *R_v_* > 95%, respectively), as accessed by Equation (1) by the integration of *π*(*A*) (expansion/compression curves) [21]:

(1) Rv=100∫πdA expansion∫πdA compression

For films of hydrophobic molecules such as the meibomian lipids, the magnitude of the hysteresis is determined by the kinetics with which the compressed surface films restore their structure and spread at the interface during area expansion [21]. Highly reversible films (Figure 3) restore their structure and spread at the interface with rate commensurable to that of the area expansion (achieved by the barriers of the Langmuir trough). Lower reversibility of the films (Figure 3, upper right panel) reflects slower reorganization and spreading of the surface films compared with the rate of area expansion. As can be seen, GG increases the surfactant properties of MGS, as manifested by the increase of *π*_max_ (the surface pressure reached at minimum surface area), but the lipid film structure is non-uniform and results in higher *π*(*A*) hysteresis (low *R_v_* = 84%), i.e., slower reorganization of the layer at dynamic blink-like compression/expansion isocycling of the film area. While the increased surfactant property of meibomian lipids may be beneficial for the stability of the tear film [22], the increased surface heterogeneity and morphological instability of TFLL is a condition typically associated with dry eye [23,24]. Therefore, the effects of GG are not straightforward for interpretation. The impact of the rest of the polymers, i.e., the formation of more uniform and thick MGS layer in their presence, aligns with the thicker and more homogeneous appearance characteristic for TFLL in healthy eyes with stable TF in vivo [1]. The slight drop of MGS isotherm reversibility in the presence of CMC and HA may be related to the increased viscosity of trough subphase into the presence of the polymers, resulting in increased friction between the film and the subphase and delayed respreading of the layers at area expansion [21].

#### 2.2.2. Effects of CHA

**Figure 4:**
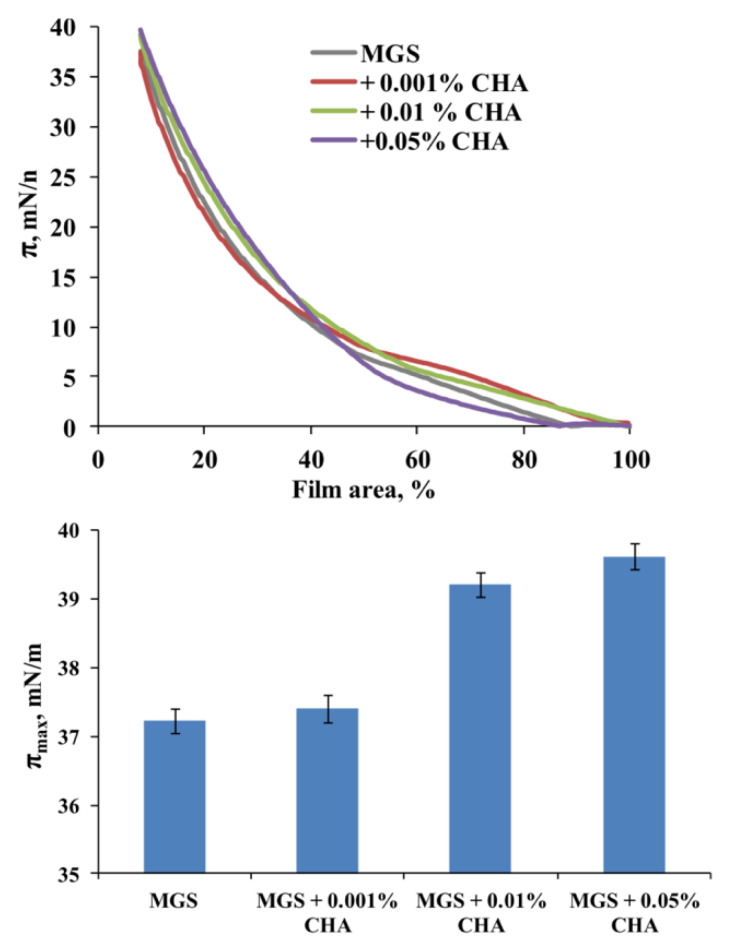
Effects of CHA on the surface properties of meibomian films. The upper panel shows the compression isotherm of meibomian (MGS) films in the presence of the polymer. The lower panel shows that CHA inclusion into the subphase results in a concentration-dependent increase of *π*_max_. The whiskers denote standard deviation (*n* = 5).

Apart from supporting the high isotherm reversibility, i.e., low surface pressure/area hysteresis loop (data in the Supplementary File, Figure S3), CHA is also able to alter the maximum surface pressure, *π*_max_, achieved at maximum compression (at lowest surface area) of the meibomian films. The data are presented in Figure 4.

**Figure 5:**
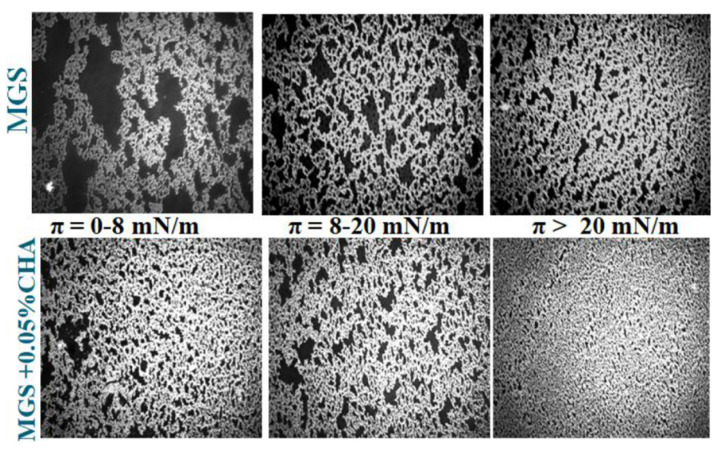
Effects of CHA on the morphology of meibomian films, as observed by BAM (image width is 500 µm).

It can be seen that CHA induced a concentration-dependent increase of *π*_max_ (a measure for the amount of meibomian lipids left at the aqueous interface at the end of compression) from 37.3 (MGS alone) to 39.5 mN/m (MGS in the presence of 0.05% CHA). The inclusion of 0.01% and 0.05% CHA resulted in a statistically significant (*p* < 0.05; one-way analysis of variance (ANOVA) with Dunnett’s post-hoc test) raise of *π*_max_ compared to “pure” MGS in the absence of the polymer. The inclusion of CHA also resulted in a more uniform and thick MGS layer spread across the air/water surface (Figure 5). The latter was manifested by the increased brightness of the BAM images and the more continuous texture of the films at *π* ≥ 20 mN/m.

Thus, it can be seen that CHA combined both potentially desirable effects. It enhanced the surfactant properties of meibum, as manifested by the increase of *π*_max_, albeit to a smaller extent than GG (see Figure 3, and also data in Supplementary File, Figures S1–S3), however in contrast with it, CHA also enhanced the surface structure of MGS films. Neither CMC nor HA altered the maximum surface pressure at minimal area of meibomian films over the entire polymer concentration range.

In a previous study, we have shown that the “standard” high molecular weight HA is able to preserve the dilatational rheological properties of meibomian films in the presence of C12—benzalkonium chloride [13]. As dilatational rheology is supposed to be critical for the “tensile strength” of the tear film, the impact of CHA on it was accessed in the next point. Control stress relaxation experiments were also performed with GG (the other MMP that altered the *π*_max_ value) and are summarized in the Supplementary File (Figures S1 and S2).

#### 2.2.3. Stress Relaxations

**Figure 6:**
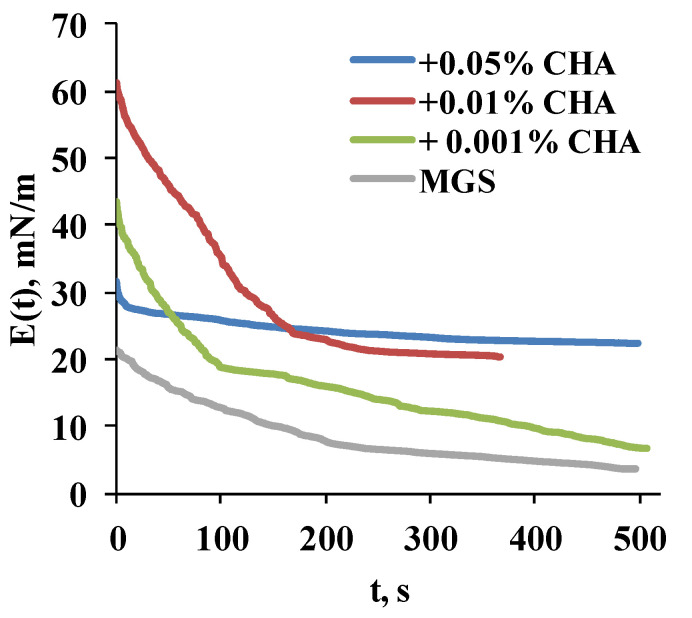
Relaxation transients of the transient elasticity modulus (mN/m) of meibomian layers, alone and in the presence of CHA.

**Figure 7:**
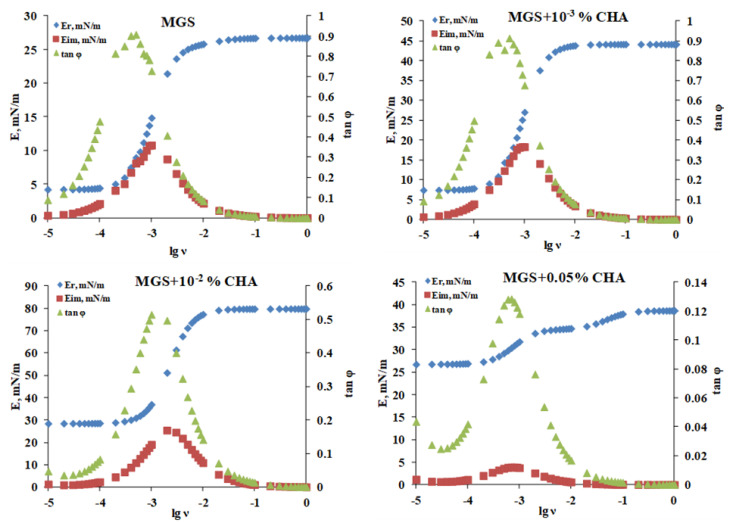
Fourier transformation analysis via Equations (3) and (4) of the stress relaxation transients of meibomian films alone and in the presence of CHA (see Figure 6).

The ∆*π*(*t*) relaxation transients were analyzed in terms of transient elasticity modulus (Figure 6) and via Fourier transformation analysis (Figure 7).

The transient elasticity modulus *E*(*t*) is calculated as:

(2) $$E\left(t\right)=A_{0}\frac{\left(d\pi \right)\;}{\left(dA\right)\;}$$

where *A*_0_ is the initial film area at which the step-like deformation *dA* is applied. The term *dπ* = (*π*_t_ − *π*_o_) accounts for the difference between the surface pressure at time *t* and the initial surface pressure prior to the step-like deformation. The *E*(*t*) approach is useful as it allows to compare the equilibrium, plateau value and elasticity reached at the completion of the relaxation.

The Fourier analysis (Figure 7) of the relaxation transients was performed as previously described [10,25] because it allows to obtain the dependence of the complex dilatational elasticity modulus, E*(*ν*), on frequency, *ν*, and to decompose it to the corresponding values of the real, *E_R_*, and of the imaginary part, *E_IM_*, of the complex modulus over the entire frequency range of 10^−5^ to 1 Hz [26,27]:

(3) $$E^{*}\left(v\right)=E_{R}\left(v\right)+iE_{im}\left(v\right)=\frac{F\left\{d∆\pi \left(t\right)/dt\right\}\;}{F\left\{dlnA/dt\right\}\;}=\frac{i6.28}{∆A/A_{0}}\int_{0}^{\infty }∆\pi \left(t\right)\exp\left(-i6.28vt\right)dt$$

where *E_R_* denotes the elasticity of the surface film, while *E_IM_* = 6.28 *νη_d_* (here *η_d_* is dilatational viscosity) accounts for the dissipative viscous properties of the film. The number 6.28 is an abbreviation of 2× Archimedes constant (2 × 3.14159…). No assumptions are made about structure of the surface film or the relaxation processes’ nature (e.g., diffusion to/from the bulk solution, molecular rearrangements, exchange with secondary adsorption layers, etc.) [26,27].

Once the real and imaginary parts of the dilatational elasticity modulus have been obtained, it is then possible to calculate the tangent of the phase angle:

(4) $$\tan\varphi =\frac{E_{IM}}{E_{R}}$$

If *E_R_* > *E_IM_*, then tan *φ* < 1 and the film is predominantly elastic. On the other hand, if *E_R_* < *E_IM_*, then tan *φ* > 1, and the film is predominantly viscous. The advantage of the Fourier analysis is that it allows to access the balance between the storage and dissipation parts of the transient viscoelastic response of the samples to the step-like deformation.

It can be seen (Figure 6) that CHA increases the transient elasticity modulus in a concentration-dependent manner, as manifested by the raise in the plateau value of *E*(*t*). It was raised from 5 (MGS alone) to 7, 23 and 28 mN/m at 0.001%, 0.01% and 0.05% CHA, respectively.

The Fourier analysis (Figure 7) revealed that CHA, in a concentration-dependent manner, raised the ratio between E_R_ and E_IM_ over the entire frequency scale of 10^−5^ to 1 Hz, which in turn resulted in a lower value of tan *φ* for meibomian films in the presence of CHA. For MGS alone tan *φ*~0.93 was realized at 10^−3^–10^−4^ Hz, while tan *φ* decreased (i.e., the ratio *E_R_*/*E_IM_* increased) to 0.52 and 0.134 at 0.01% and 0.05% CHA, respectively. The enhanced elasticity is known to correlate with the stability of the TFLL morphology between blinks and with the overall stability of TF in vivo [2,12,23].

It should also be noted that the shape of the *E*(*t*) relaxation transients and of Fourier images of the relaxations is significantly modified between meibum alone and in the presence of CHA, as well as between the films at the different polymer concentrations. The latter effect indicates that the CHA-induced changes in MGS morphology also alter the arrangement of the molecules primarily responsible for the elastic component of the relaxation, i.e., the polar lipids located at the TFLL/aqueous interface. This aligns with the proposed mechanisms of rapid polar (H-bonding) and non-polar (hydrophobic) interactions of the MMP moieties with the polar lipids headgroups and the adjacent carbonyls [13,15].

## 3. Discussion

It can be seen that among all the polymers studied, only GG is not capable to form fine-structured ferning patterns by itself, and also, it is not able to enhance the inherent capability of tears to do so. This might be related to the propensity of GG to form highly hydrated viscous gel at physiological ionic strengths [28]. It is reported that the resistance of the polymer moieties to the evaporative loss of hydration water impairs the MMP capability to form ferning patterns [29]. Furthermore, the strong interaction of GG with self-same molecules might be energetically preferred compared to the GG interaction with the secretory mucins in the tear fluid, which in turn will perturb the fine microstructure of the tear ferns. The strong cohesion between the molecules in the GG gel is expected to result in higher viscosity of the PBS subphase beneath the meibomian layers, and thus stronger friction between the film and the subphase even at the high shear rates realized in the course of blink-like isocycling. In turn, the latter effect hampers the isothermal reversibility and the uniform spreading of the lipid layer [30]. Thus, GG-based ophthalmic formulations might need careful optimization of their composition (in terms of polymer’ molecular weight or concentration) in order to achieve optimal clinical efficiency. A possible strategy is to decrease the molecular weight of the GG polymer chain, which should lead to (i) lower strength of cohesion between self-same GG molecules and enhanced interaction with the tear fluid MUC5AC, thus enhancing the formation of properly structured tear ferns, and (ii) modified interplay with the functionality of the meibomian film [12].

The rest of the MMP showed good capability to form post-evaporative ferning patterns by themselves alone and also to enhance the density of the fine microstructure of the ferning patterns of natural human tears. It is thought that good miscibility of MMP with the tear secretory mucins will result in enhanced ferning patterns of the MMP/tear mixture [19,20]. The latter is in turn an indicator of the quality of the mucoaqueous gel. Therefore, it is supposed to report for enhanced “tensile strength” of the tear film [31], i.e., enhanced capability of TF to resist the stretching and the thinning caused by the capillary suction pressure exerted by the upper and lower eyelid menisci at the interblink in an open eye. Particularly impressive in terms of ferning pattern formation properties was CHA, which not only showed excellent ability to do so (alone or included into tears) but also displayed formation of post-evaporative structured precipitates at 0.001%, i.e., an order of magnitude lower than HA and CMC.

Apart from influencing the structure of the post-evaporative ferning patterns, MMP also had a noticeable effect on the surface properties and on the morphology of the meibomian layers at the air/water interface. Once again, GG was distinct from the rest of the MMP as it resulted in significantly increased surfactant properties of MGS (raise of *π*_max_), which, however, came with the price of compromised, heterogeneous surface morphology, and decreased isotherm reversibility (increased hysteresis loop). Still, similarly to CHA (i.e., the other MMP that was able to raise *π*_max_ achieved at minimum surface area), it was able to maintain the predominantly elastic properties of the meibomian films (see Supplementary File, Figures S1 and S2). While the increased surface activity may contribute towards the stabilization of the air/tear interface, the heterogeneous structure and the increased hysteresis (indicating increased energy dissipation in the course of blink-like deformations) are features typical for films by lipid samples obtained from donors with meibomian gland disease [22,23,24]. The rest of the MMP showed capability to enhance the spreading of meibomian lipids, as can be seen by the polymer-induced formation of thicker and more uniform lipid layer morphologies, a feature characteristic for TFLL structure in the case of stable TF in healthy eyes [23,24].

This spreading enhancement effect can be assigned to the formation of interfacial gel-like networks due to H-bonding of the polymer moieties, with each other and with the polar lipids headgroups, leading to increased film rigidity, more even two-dimensional (2D) distribution of the lipids and water incorporation into TFLL [13,15]. This mechanism takes place on the physiologically relevant timescale of seconds, which emphasizes that all the layers of TF should be viewed not as isolated structures but as the parts of an integral system in continuous interplay among themselves.

The importance of mucin-like polymers for the performance of TFLL was confirmed in vivo by the capability of a 0.1% HA-induced increase (from <60 to 75 nm) of TFLL thickness of lipid-deficient individuals [16], and by the comparable effect for >1 h post-instillation of 3% diquafosol ophthalmic solution (a drug promoting the rapid secretion of MUC5AC) on the TFLL in healthy human eyes [17].

The interaction of CHA with meibomian films again stood out, and thus it was characterized in greater detail as this also allows comparing the polymer effects with the impact of HA on meibomian lipids that was studied earlier [13]. It can be seen that CHA, in a concentration-dependent manner, raised the surfactant properties of the MGS layer (raise of *π*_max_), while at the same time, the isothermal reversibility remained high (i.e., the hysteresis remained low). As revealed by the stress relaxation studies, the presence of CHA also raised the relative contribution of the real part of the complex dilatational modulus of the meibomian films. The effects of HA observed in our previous study were similar but (i) they needed higher (~0.1%) concentration to take place and (ii) CHA does not alter *π*_max_. These data are in excellent agreement with the information about the differences in the interactions of CHA and HA with the lipids of stratum corneum (long-chain hydrophobic molecules partially similar to the ones found in meibum) [32]. It is found that CHA penetrates faster and binds stronger to synthetic lipid membranes, probably due to the smaller size of the CHA molecule compared to HA with the same molecular weight, which is due to the cross-linking. Also, it was reported that CHA gels are more elastic compared to HA gels due to the cross-linking-induced change in the pattern of H-bonds and hydrophobic intra- and inter-molecular interactions [33,34].

Although our work is essentially centered on the biophysical/physical chemistry aspects of therapeutic action, certainly, mucomimetic polymers can exert their effects as dry eye therapeutics also via biochemical routes [35]. These effects are particularly well-studied for high molecular weight hyaluronic acid species, as these are widely used in artificial tears to treat dry eye symptoms. Because of its size and viscosity, HA enhances the hydration of the ocular surface and minimizes its friction with the eyelid wiper at blink [36]. It is shown in Chang conjunctival cell lines that HA suppresses the production of reactive oxygen species (estimated via dichlorofluorescein diacetate and hydroethidine tests) while also displaying cytoprotective effects by reducing the toxicity of eyedrop preservatives [37]. Similar outcomes were also reported in animal studies [38]. Furthermore, high molecular weight HA displays anti-inflammatory and immunosuppressive properties, and is considered one of the key players in the tissue regeneration process [39]. All these activities are exerted via signaling pathways involving the interaction of HA with a plethora of key receptors: LYVE-1: lymphatic vessel endothelial receptor 1, CD44: antigen, a type of transmembrane glycoprotein, RHAMM: receptor for hyaluronan-mediated motility, and TLR-4: toll-like receptor-4 [40]. Numerous studies have shown that HA signaling plays an important role in angiogenesis regulation, mainly by influencing endothelial cell behavior [39]. It is interesting that the HA physiological effects are heavily dependent on the polymer molecular weight (Mw). While HA with Mw ≥ 200 KDa are beneficial for the cellular health, low molecular weight HA is a potent proinflammatory molecule. This phenomenon is attributed to the different manner of interaction with the HA receptors, CD44 and RHAMM in particular [41]. It is thought that these diverse effects stem from the impact of the molecular weight on the HA conformation: large >MDa HA is a random coil, while very small (e.g., 10 kDa) HA behaves like a rod. Size exclusion chromatography and multiangle light scattering studies revealed that HA mass-to-diameter ratio showed a transition in the 150–250 kDa size range (~65 nm). Hence, the HA rod-to-coil transition occurs in the size range that specifically activates cell signaling by the receptors discussed above. Thus, size-specific signaling could be due to (i) unique external receptor/HA conformation changes enabling transmembrane-mediated activation of cytoplasmic domains, or (ii) transition-size HA may enable multiple receptors to bind the same HA, creating new internal signal-competent cytoplasmic domain complexes [42]. A major limitation of HA eyedrops is that their viscosity decreases as a function of time, which in turn decreases the formulation’s activity and effectiveness in dry eye treatment [43]. It is one of the reasons that motivated the implementation of cross-linked HA (CHA) as a more viscoelastic material, which enhances the contact time with the ocular surface, and displays better durability than the linear form [44].

Thus, although the biophysical and mechanistic effects are thought to be the primary route of action of mucomimetic polymers in the restoration of the tear film and ocular surface integrity, the biochemical interactions may also significantly contribute, especially in cases where long-term residence time of the polymers at the ocular surface is achievable [1].

Another characteristic limitation of the biophysical studies of tear and meibomian samples is the small sample size (i.e., the small number of volunteers from which the specimens were collected). Typically, samples are either from a single donor, or pooled from a small number of volunteers (as done in the current study) [1,2,9,10,11,12,13,14,25,30,38]. Still, the studies of mutually independent teams collecting samples from vastly different (in terms of age, sex, ethnicity, etc.) donors, confirmed the number of features characteristic for healthy tear and meibum, i.e., ferning pattern formation and the formation of continuous and rough viscoelastic duplex films of multilayer thickness at the air/tear surface. This agreement between the results of different groups suggests that in spite of the small sample size of laboratory biophysical studies, the key tear and meibum properties that are registered and analyzed are indeed reproducible and representative.

In conclusion, it can be said that polyanionic non-surface-active polysaccharides resembling the sialic acid residues of the tear secretory mucins may influence both (i) the structure of the mucoaqueous gel in the AT (responsible for the “tensile strength” of the TF at interblink in an open eye) [31], and (ii) the spreading and the structure of the tear film lipid layer [12]. Such endogenous and exogenous MMP may play a key role for the relationship and synchronization between the TF layers, ensuring its normal functionality in agreement with the recent findings about the enhanced TFLL thickness and uniformity after the instillation of diquafosol sodium, a MUC5AC secretagogue. With this work, we hope to foster the attention for the need of further in-depth studies of such effects.

## 4. Materials and Methods

### 4.1. Materials

Human meibum and tears were collected by Prof. Norihiko Yokoi from Kyoto Prefectural University of Medicine in accordance with the tenets of the Declaration of Helsinki and with the permission of the Ethics Committee. All healthy volunteers, three females (25–43 years old) and one male (33 years old), that served as donors signed an informed consent form. The meibomian lipids from healthy volunteers were collected by the “soft squeeze” method [1,2], while tears were collected by the capillary tube method [19]. Both meibum and tears were stored as pooled samples to ensure sufficient amount of material for multiple experiments. All the mucomimetic polymers were donated by Santen SAS Pharmaceuticals, France: hyaluronic acid (HA) with mean molecular weight (MMW) of 1 MDa (polydispersity index < 1.2; Echelon Biosciences, Salt Lake City, UT, USA), cross-linked hyaluronic acid (CHA) with MMW 2.5 MDa and 30% cross-linking degree (cross-linking with 1-ethyl-3-(3-dimethyliaminopropyl) carbodiimide hydro-chloride) (BIOITECH S.r.l.; Roma, Italy), carboxymethyl cellulose (CMC) with MMW 0.7 MDa (Sigma-Aldrich, Saint Louis, MS, USA) and gellan gum (GG) with MMW 1 MDa (Sigma-Aldrich, Saint Louis, MS, USA). These MMP were selected on the basis of their implementation for ophthalmic use [35].

### 4.2. Methods

#### 4.2.1. Ferning Patterns

Ferning patterns were obtained after 10 min and were allowed for the evaporation of 1–2 µL droplets of MMP or tear/MMP solutions at 60% relative humidity on clean microscope glass. The patterns were observed with a 10× digital microscope (Olympus DP72).

#### 4.2.2. Langmuir Surface Balance Studies

##### Compression Isotherms

Surface pressure/area (*π*-*A*) isotherms were measured [10] by Langmuir surface balance µ Trough XS, area 135 cm^2^, volume 100 mL (Kibron, Helsinki, Finland), via the Wilhelmy wire probe method (instrumental accuracy 0.01 mN/m). Physiological saline solution buffer (PBS, pH 7.4) was utilized as trough subphase. A micro-syringe (Hamilton Co., Reno, NV, USA) was used to uniformly spread human MGS (35 µL of 1 mg/mL chloroform solution) over the air/saline surface. The trough was fitted under an acrylic cover to protect the surface from dust and to suppress the PBS subphase evaporation (90% relative humidity is maintained under the cover). After 15 min were provided for chloroform evaporation, film compression was initiated by two symmetrically moving barriers. Dynamic compression–expansion isocycling of the film area was performed at the maximum barrier’s rate (70 mm/min), at which there was no leakage of the film. Ten consecutive cycles were performed with each film studied. Typically, after the third cycle, the *π*(*A*) curves attained constant shape and those *π*(*A*) isotherms were analyzed further. All isotherms were repeated at least in triplicate, and the difference between the repetitions was less than 2%. The experiments were performed at 35 °C, i.e., the physiological temperature of the ocular surface. The films’ morphology was monitored by a Brewster angle microscope (MC-BAM, Imperx, Boca Raton, FL, USA).

##### Stress-Relaxation Studies via the Small Deformations Method

The dilatational viscoelasticity of meibum films was accessed by measurement of the surface pressure relaxation transients after a small rapid compression step was applied to the layers. First, the film was compressed to initial surface pressure, *π*_0_ = 15 mN/m. Then, the film was instantaneously contracted with a small compression step, ∆*A*/*A*_o_ = 5% ± 1% (*A*_o_ is initial film area, and ∆*A* is area change). The dependence of the real, E_R_, and imaginary part, E_IM_, of the complex dilatational elasticity modulus, E*(*ν*), on frequency, *ν*, was accessed via Fourier transformation, F, of the relaxations [27,45,46], performed as previously described [26,27,46], by commercial Fourier transform software provided by Kibron Inc. (Helsinki, Finland).

## Data Availability

The data presented in this study are available in this study and in the associated Supplementary Materials.

## Supplementary Materials

The following are available online at https://www.mdpi.com/1422-0067/22/5/2747/s1.
