# Supplementary material for: Interactions of Meibum and Tears with Mucomimetic Polymers: A Hint towards the Interplay between the Layers of the Tear Film
Source: Int J Mol Sci. 2021 Mar 9;22(5):2747. doi: 10.3390/ijms22052747 (PMC7963170; doi:10.3390/ijms22052747)
Supplement: Supplementary file 1 [file ijms-22-02747-s001.pdf]

Supplementary file

I. Stress relaxation studies with gelan gum

This point briefly summarizes a control stress relaxation experiment with gelan gum. The experiment shown is at 0.05% the minimum concentration at which microdroplets of GG solution formed structured post-evaporation precipitates. At 0.1% GG solutions become too viscous and it was not possible to perform Langmuir surface balance studies at this concentration.

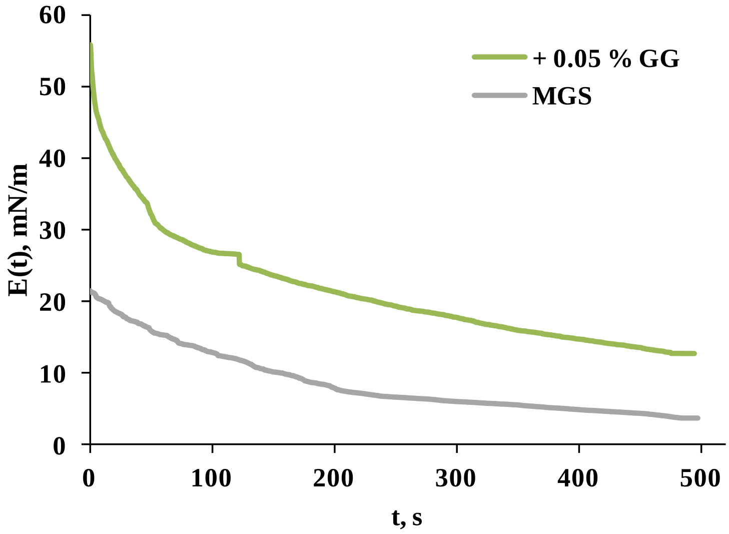

Fig. S1. The stress relaxation transient of MGS, pure and in presence of 0.05% GG.

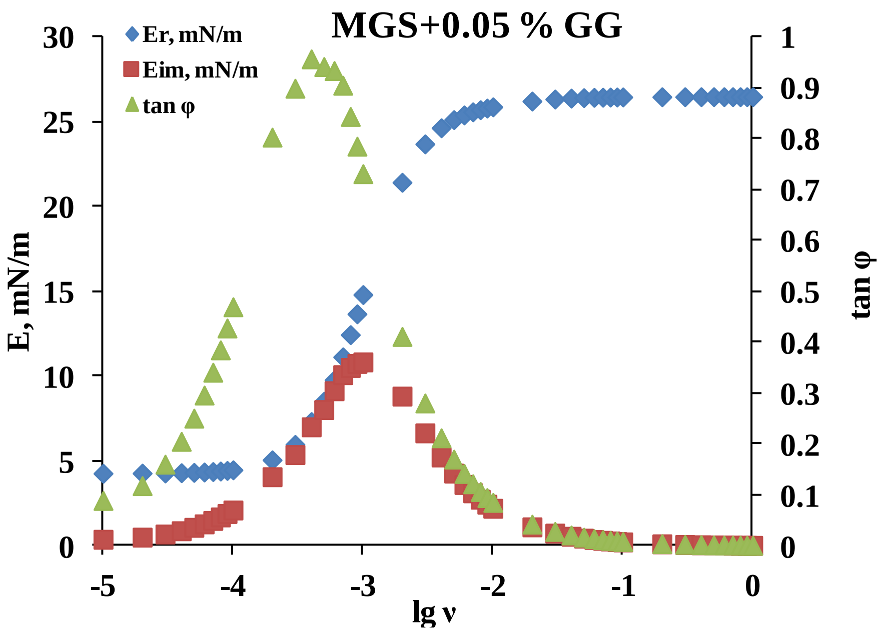

Fig. S2. Fourier analysis of the relaxation transient shown at Fig. S1.

As can be seen similarly to CHA (i.e. the other MMP that was able to raise the maximum surface pressure realized at the end of film compression), the inclusion of GG maintained the predominantly elastic surface properties of the meibomian films.

II. Compression/expansion isocycles of meibomian films in the presence of cross-linked hyaluronic acid

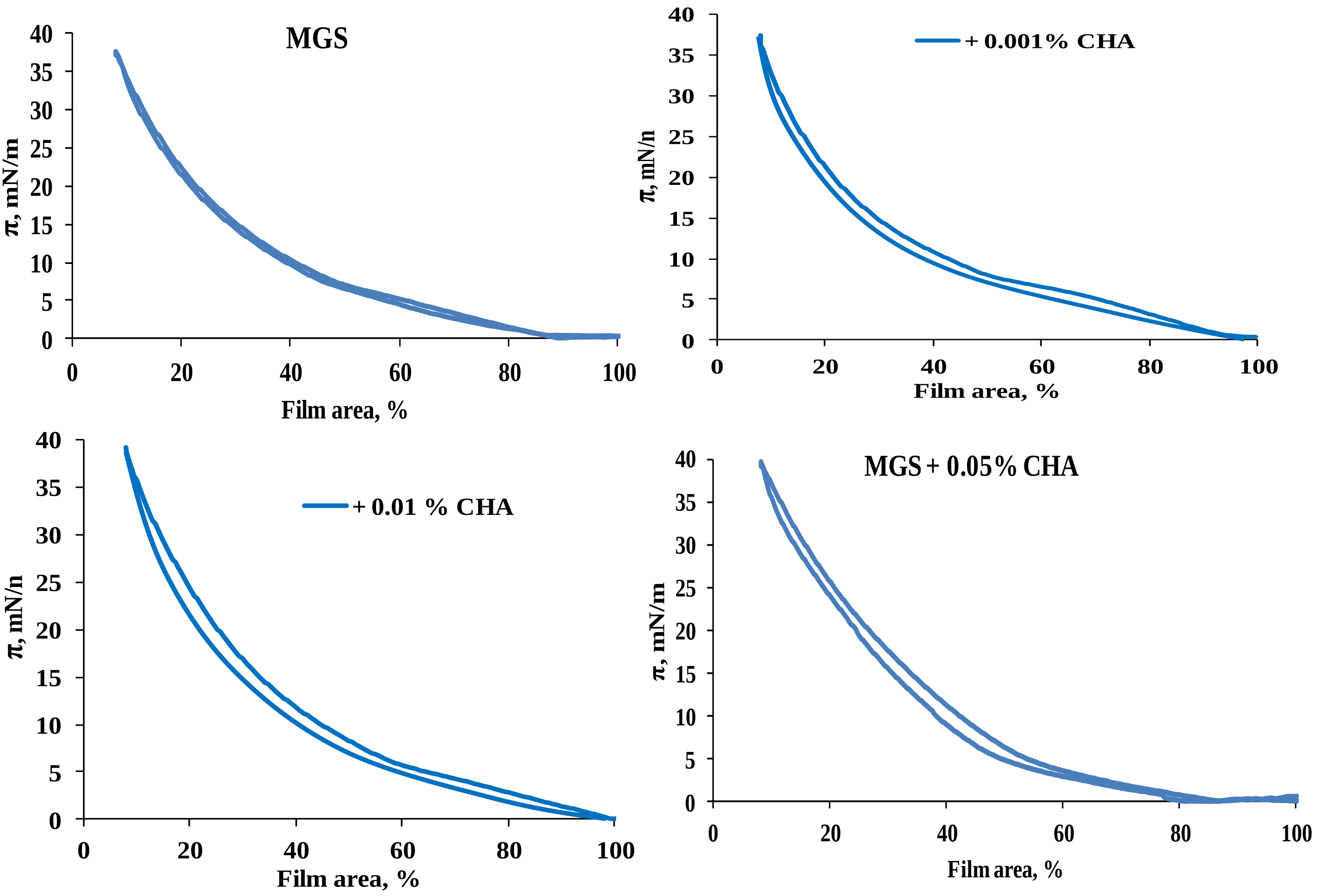

Fig. S3. Compression expansion isocycles of MGS layers, alone and in presence of denoted concentrations of CHA.

It can be seem that although the presence of CHA resulted in a slight drop of isothermal reversibility (Rv), still Rv remained >95% for all the polymer concentrations. At 0.1 % CHA the subphase become too viscous and it was not possible to conduct reproducible experiments.
